# Comment on Cancello et al. Sarcopenia Prevalence Among Hospitalized Patients with Severe Obesity: An Observational Study. *J. Clin. Med.* 2024, *13*, 2880

**DOI:** 10.3390/jcm13226685

**Published:** 2024-11-07

**Authors:** Sabine Schluessel, Katharina Mueller, Michael Drey

**Affiliations:** Department of Medicine IV, University Hospital, Ludwig Maximilian University Munich, Ziemssenstraße 5, 80336 Munich, Germany

**Keywords:** sarcopenic obesity, skeletal muscle mass, fat mass, handgrip

## Abstract

Dear Editor, we read the article “Sarcopenia Prevalence Among Hospitalized Patients with Severe Obesity: An Observational Study” and found it to be of great interest. The exploration of this important topic is highly commendable; however, we would like to highlight a critical issue that has not been fully addressed in this study. Specifically, the study does not fully adhere to the consensus definition of sarcopenic obesity (SO) as outlined by the European Society for Clinical Nutrition and Metabolism (ESPEN) and the European Association for the Study of Obesity (EASO), as we will explain below.

## 1. Introduction

Sarcopenic obesity (SO) is defined by the coexistence of obesity and reduced muscle mass and function [1]. This condition poses a serious challenge for healthcare systems due to its strong association with increased morbidity and mortality [2]. In 2022, ESPEN and EASO published updated guidelines for diagnosing SO [3]. According to data from the KORA-Age study, the prevalence of sarcopenic obesity in Germany among individuals aged 65 and older is approximately 4.5%, with men being slightly more affected (5%) compared to women (4%) [4]. The study also revealed that sarcopenic obesity is significantly linked to cognitive decline [4]. Dementia and mild cognitive impairments are far more common in individuals with SO compared to those who have either sarcopenia or obesity alone [5]. Additionally, sarcopenic obesity has been associated with a higher risk of comorbidities such as cardiovascular disease and arthritis, along with multimorbidity, polypharmacy, and an elevated mortality rate [3]. This suggests that sarcopenia and obesity have compounded negative effects, with mortality rates being higher in individuals with SO than in those suffering from sarcopenia or obesity alone [6]. Therefore, the accurate diagnosis of sarcopenic obesity is crucial for clinical practice and public health.

## 2. How Is Sarcopenic Obesity Diagnosed?

**Figure 1:**
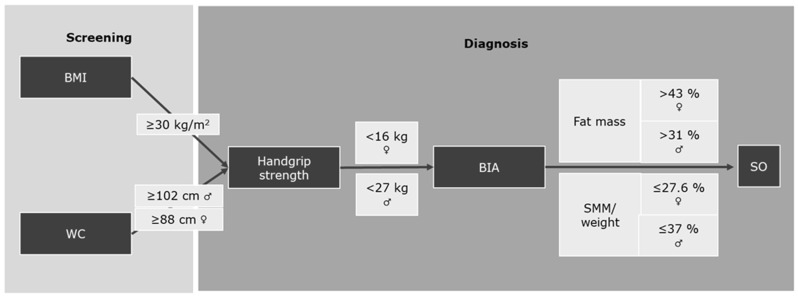
Cut-off-scores for a Caucasian cohort aged over 60 years. WC: waist circumference; BMI: body mass index; BIA: Bioelectrical Impedance Analysis; SMM: skeletal muscle mass; SO: sarcopenic obesity.

Diagnosing sarcopenic obesity involves three key measurements—body mass index (BMI), handgrip strength, and whole-body composition—which are typically assessed using Dual-Energy X-ray Absorptiometry (DXA) or Bioelectrical Impedance Analysis (BIA) [3]. These tools allow for the precise evaluation of both muscle mass and fat mass, providing a clearer diagnosis. In 2022, the diagnostic criteria for sarcopenic obesity were revised according to recommendations from ESPEN and EASO [3]. One of the most significant changes was the shift from normalizing muscle mass relative to height squared (as was previously carried out) to normalizing it relative to body weight. This change was made because the former method often overestimated muscle mass in obese patients. For non-obese individuals, muscle mass continues to be normalized to height. The cut-off values for fat mass and muscle mass vary depending on ethnic population and age group [3]. For instance, specific diagnostic algorithms for Caucasian individuals over the age of 60 are illustrated in Figure 1 for BIA. Thresholds for other age groups, DXA or ethnic populations can be found in the supplement of the ESPEN and EASO consensus publication [3].

## 3. High Fat Mass and Reduced SMM/Weight Are Necessary for the Diagnosis of SO

The consensus definition provided by ESPEN and EASO clearly states that in the final step of diagnosing sarcopenic obesity (SO), both low skeletal muscle mass relative to weight (SMM/weight) and high fat mass must be included [3]. This is also evident in the flowchart of the consensus statement, which serves as the central figure of the original publication of ESPEN and EASO. However, in the article “Sarcopenia Prevalence Among Hospitalized Patients with Severe Obesity: An Observational Study”, Cancello et al. completely overlooked this critical step by not including fat mass in their diagnostic process, which might have led to an overestimation of the SO prevalence [7]. Fat mass is crucial for identifying the “obesity” component of SO. Simply using BMI as a screening tool, as outlined by ESPEN and EASO, is insufficient for a proper diagnosis. In Figure 1 of Cancello et al.’s original publication, their flowchart shows that they only focused on strength deficits and muscle mass deficits, while neglecting fat mass percentage [7]. This omission is significant, as fat mass data should have been available to the authors and could have easily been incorporated to correct the missing parameter. Without this, their diagnostic algorithm does not fully adhere to the ESPEN and EASO criteria. Cancello et al. also reported that they included different age groups ranging from 18 to 90 years old. The appropriate cut-off values for fat mass, which they omitted, are provided in the supplement of the ESPEN and EASO consensus statement [3]. Specifically, for individuals aged 20–39 years, the fat mass threshold is >39% for women and >26% for men. For the 40–59 years group, the cut-offs are >41% for women and >29% for men, and for individuals aged 60–79 years, the cut-offs are >43% for women and >31% for men. We therefore strongly recommend that the authors repeat their analysis, incorporating fat mass as per the consensus criteria, and update Figure 1 in their original publication to reflect this adjustment. This will ensure that their findings are consistent with established guidelines and provide more accurate prevalence data.

## 4. Conclusions

In conclusion, while the study by Cancello et al. addresses an important and relevant topic, it overlooks a key component in the diagnosis of SO by failing to incorporate fat mass in its diagnostic process. The consensus definition provided by ESPEN and EASO clearly states that both low skeletal muscle mass relative to body weight and high fat mass are required for a proper SO diagnosis. This omission likely led to an overestimation of the prevalence of SO in their study. We strongly recommend that the authors revise their analysis to include fat mass, using the appropriate cut-off values provided in the ESPEN and EASO guidelines. By making this adjustment and updating their diagnostic flowchart, their findings would more accurately reflect the true prevalence of sarcopenic obesity and adhere to current consensus criteria. This revision is essential to provide reliable data that can be effectively used for clinical and public health purposes.
